# Effects of Chromium Yeast Supplementation on Serum *hsp60* and *hsp70*, mRNA Expression in Heat-Stressed Lambs

**DOI:** 10.3390/vetsci12090801

**Published:** 2025-08-24

**Authors:** Edwin Sandoval-Lozano, Iang S. Rondón Barragán, Andrés Sandoval-Lozano, Román David Castañeda-Serrano

**Affiliations:** 1 Research Group in Livestock Agroforestry Systems, Faculty of Veterinary Medicine and Zootechnics, Department of Animal Production, University of Tolima, Ibagué 730006, Colombia; esandovall@ut.edu.co; 2 Research Group in Immunobiology and Pathogenesis, Faculty of Veterinary Medicine and Zootechnics, Department of Animal Health, University of Tolima, Ibagué 730006, Colombia; isrondon@ut.edu.co; 3 Interdisciplinary Research Group in Tropical Fruit Cultivation, Faculty of Agronomy, University of Tolima, Ibagué 730006, Colombia; sc2salol@uco.es

**Keywords:** heat stress, cortisol, heat shock proteins, qPCR, transports of glucose, organic chromium

## Abstract

Small ruminant production is increasingly affected by heat stress, with recent heat waves highlighting growing economic and welfare-related challenges. Chronic exposure to elevated temperatures disrupts thermoregulation, reduces feed intake, slows growth, compromises meat quality, and increases mortality. This study evaluated the effects of chromium-yeast supplementation at different doses and timepoints on physiological and molecular stress biomarkers in heat-stressed lambs. Forty-eight clinically healthy 6-month-old Katahdin lambs (average weight 20 ± 2.9 kg) were assigned to a 2 × 4 factorial design, with two ambient temperature conditions (heat stress [HS] and thermoneutral [TN]) and four levels of dietary Cr-yeast (0, 0.2, 0.4, and 0.8 mg/kg of dry matter intake). Lambs were housed individually in pens (1.2 × 2.5 m), with ad libitum access to water, and fed a 50:50 corn silage and concentrate diet (excluding mineral premix) twice daily. Blood samples were collected at days 0, 30, and 60 to evaluate plasma cortisol and the expression of *hsp60* and *hsp70*. Chromium bioavailability was assessed by blood levels using absorption chromatography, and glucose clearance was measured at the end of the experiment. Significant reductions in cortisol and *hsp70* expression were observed after 30 days of Cr-yeast supplementation under HS conditions (*p* < 0.05), particularly at the highest dose. For *hsp60*, a significant reduction was observed at the highest dose on day 30 under HS (*p* < 0.05). These effects were not sustained on day 60 (*p* > 0.05). No significant differences were detected under TN conditions (*p* > 0.05). These findings suggest that Cr-yeast may offer short-term physiological and cellular protection against chronic heat stress in lambs.

## Simple Summary

Heat stress in lambs can cause significant damage to their health and performance, primarily affecting growth. During periods of high temperatures, sheep experience an increase in cortisol levels, leading to an imbalance in cellular homeostasis and the expression of heat shock proteins (*hsp*), such as *hsp60* and *hsp70*. These proteins are crucial for cellular protection, but their overexpression indicates cellular damage. Supplementing with chromium is important, as it is associated with glucose transport, antioxidant activity, and a potential reduction in stress. Implementing this supplementation can contribute to improved animal welfare and increased productivity in sheep farming.

## 1. Introduction

Sheep production contributes to global food systems and environmental sustainability [1,2,3]. In some regions, it supports rural economies by providing income and food security for small-scale farmers and indigenous communities [4,5].

However, rising temperatures and frequent heat waves become serious challenges to their welfare and productivity, particularly due to heat stress (HS) [6,7,8]. The chronic HS can impair thermoregulation, leading to reduced feed intake, growth rate, and meat tenderness, also increasing the mortality of animals [7,9,10].

At the cellular level, HS triggers the expression of heat shock proteins (*hsp*), which support protein folding and stress adaptation [11,12,13]. *hsp70* protein plays a key role in oxidative stress management and cardiovascular regulation, has been widely studied in ruminants, such as goats [14], buffalo [15], dairy cows [12], and sheep [16]. Structurally, *hsp70* comprises three domains that form a substrate binding pocket and lid configuration [17,18,19,20]. However, its folding efficiency may be compromised in crowded intracellular environments, where *hsp60* complements its function by enhancing thermos-tolerance, facilitating protein refolding, mitochondrial import, and degrading misfolded proteins [21,22,23,24,25]. Additionally, cortisol increases under chronic stress and serves as a reliable physiological biomarker, promoting gluconeogenesis, protein catabolism, and fat breakdown to meet energy demands [26]. It is regulated by the HPA axis and rises during acute stress [27,28,29].

Among the strategies to mitigate HS, organic chromium has gained attention due to its potentially greater bioavailability [30] and its capacity to improve stress tolerance by modulating metabolic responses, including improved feed intake, enhanced respiratory rates, and better glucose clearance [26,31,32,33,34,35]. However, organic chromium requirements for lambs remain unclear [36], and its bioavailability is still not well understood [37]. While organic chromium supplementation shows promise responses, inconsistent reports in lambs have been observed, especially across a wide dosage range [30], and notably, studies reporting potential structural effects and toxicity have primarily involved chromium picolinate [38], whereas chromium chelated in yeast (Cr-yeast) appears safer while offering rumen-related benefits, particularly at doses below 0.8 mg/kg [30,39,40].

Some differences across the evaluation period were observed under HS; lambs show clear HS responses within 1 to 30 days, highlighting early physiological changes [41,42]. While chronic and adaptive effects appear around 60 days [41,43]. Therefore, this study aimed to evaluate the effects of Cr-yeast supplementation on stress biomarkers (*hsp60*, *hsp70*, and cortisol) in heat-stressed lambs, testing different doses (0, 0.2, 0.4, and 0.8 mg/kg) and two time periods (30 and 60 days) to identify an optimal level for improving stress resilience and animal welfare.

## 2. Materials and Methods

### 2.1. Experimental Animals

This study was conducted in accordance with the guidelines established by the Department of Bioethics and Animal Welfare of the University of Tolima (Approval No. 10, 2023). A total of 48 non-castrated clinically healthy 6-month-old Katahdin lambs (average weight 20 ± 2.9 kg) were used in a 60-day factorial experiment (2 × 4), involving two environmental conditions—heat stress (HS) and thermoneutral (TN)—and four levels of Cr-yeast supplementation. The Cr-yeast Bio-Chrome™ (Alltech^®^, São Pedro do Ivaí, Paraná, Brazil) contained 1000 ppm of chromium, equivalent to 1 mg of chromium per gram of product. The supplementation levels were 0, 0.2, 0.4, and 0.8 mg of chromium per kg of dry matter intake (DMI), with six animals assigned to each treatment. To ensure complete consumption, the Cr-yeast was administered daily in gelatin capsules.

Before the trial began, all lambs were individually weighed, dewormed, and fed during a 12-day adaptation period. For both HS and TN conditions, the temperature–humidity index (THI) was calculated using the following formula [44,45]:

*THI* = (1.8 × *T*° + *32*) − (0.55 − 0.55 × *RH*/*100*) × (1.8 × *T*° − 26).

where *T*° represents the ambient temperature (°C) and *RH* is the relative humidity (%).

Based on established *THI* scales for small ruminants, a THI 69 ± 3 is considered TN, and 80 ± 3 denotes moderate to severe HS [46,47]. The HS condition was induced using flame-based heaters, activated three times daily, and achieving an average temperature of 38 ± 1 °C and a THI of 86.40. In the TN condition, the temperature was maintained at approximately 28 ± 1 °C, with a THI of 72.30. Temperature and humidity were continuously recorded using a HOBO^®^ U23 Pro v2 device (Onset Computer Corporation, Bourne, MA, USA). Lambs were housed individually in pens measuring 1.2 × 2.5 m and had ad libitum access to water. Animals were fed twice daily (0800 and 1600 h) with a diet composed of 50% corn silage and 50% concentrate, excluding a mineral premix (Table 1).

### 2.2. Determination of Chromium in Blood

Before the daily supplementation, to determine the availability of chromium in blood, animals received a single dose of each level of Cr-yeast. Blood samples were collected by jugular venipuncture at 0, 3, 6, 12, 24, 36, and 72 h to monitor changes in blood chromium concentration. Samples were collected into vacuum tubes without anticoagulant, and whole blood was used for chromium determination to maximize analytical accuracy. After collection, samples were stored at −20 °C until analysis. Blood samples were prepared for analysis by wet ashing with trace metal grade nitric acid (Fisher Scientific, Waltham, MA, USA). Chromium concentration was determined by atomic absorption spectrophotometry with a graphite furnace, using a Perkin Elmer model 2380 spectrophotometer equipped with an HGA-400 graphite furnace and AS-40 autosampler (PerkinElmer Inc., Waltham, MA, USA). The analysis was performed following the methodology described by Spears et al. [37] and Bermejo-Barrera et al. [48].

### 2.3. Sample Collection for qPCR and Cortisol

After the sampling for availability, the animals were fed daily with the doses of chromium. At 30 and 60 days after supplementation, samples of blood were taken via jugular venipuncture from each animal, for a total of 96 samples. The catheter was flushed with sterile saline and heparin (diluted to 10 IU/mL) between samples. Blood samples were preserved at −20 °C in heparinized tubes for qPCR and in serum tubes for cortisol metabolites.

### 2.4. RNA Extraction, cDNA Synthesis and Gene Expression Analysis

Total RNA was extracted from blood samples using the RNA-Solve^®^-chloroform protocol (Omega Bio-tek Inc., Norcross, GA, USA), an adapted method based on the guanidinium thiocyanate-phenol-chloroform extraction technique [49,50]. The concentration and purity of the extracted RNA were determined spectrophotometrically using the NanoDrop^TM^-One (Thermo Fisher Scientific Inc., Waltham, MA, USA) by measuring absorbance at 260 and 280 nm, and integrity was verified by the 260/280 ratio, with values between 1.8 and 2.0 considered acceptable [51,52]. RNA samples were homogenized to 200 ng/μL, and cDNA was synthesized using the GoScript™ Reverse Transcriptase kit (Promega Corporation, Madison, WI, USA). following the manufacturer’s guidelines. The relative expression of *hsp60* and *hsp70* genes was measured by quantitative real-time PCR (qPCR) in a QuantumStudio^TM^ 3 real-time PCR system (Applied Biosystems, Thermo Fisher Scientific Inc., Waltham, MA, USA), by fast ramp program, using the Luna^®^ Universal qPCR Master Mix kit (New England BioLabs Inc., Ipswich, MA, USA). All reactions were performed in triplicate. The relative gene expression was normalized using the Tyrosine 3-monooxygenase/tryptophan 5-monooxygenase activation protein, zeta polypeptide (*Ywhaz*) as a reference gene. The data obtained were analyzed using the 2^−ΔΔCt^ method [53] and were expressed as fold-changes compared to the expression of the reference gene. Primer sequences for *hsp60* and *hsp70* amplification are shown in Table 2.

Thermal cycling conditions were an initial denaturation for 1 min at 95 °C, then 40 cycles of denaturation for 3 s at 95 °C, and annealing for 30 s at 60 °C. Subsequently, a melting step was performed at 95 °C for 1 s, 60 °C for 20 s, and a continuous rise in temperature to 95 °C at a rate of 0.15 °C per second [51].

### 2.5. Cortisol Analysis and Glucose Clearance Test

Enzyme-linked immunosorbent assay (ELISA) was performed using AccuBind^®^ (Monobind Inc., Lake Forest, CA, USA) 25 µL of each sample were added to antibody coated wells along with enzyme-labeled cortisol following the manufacturer’s recommendations. Absorbance was measured at 450 nm, showing an inverse relationship with cortisol concentration.

For glucose clearance, after 60 days of supplementation, animals were fasted and glucose was administered intravenous at 0.45 g/kg of BW^0.75^, according to the methodology suggested by Haldar et al. [35]. Blood samples were collected at 0 and at 20 min intervals thereafter until basal glucose levels were reestablished. Glucose concentrations were expressed in mg/dL.

### 2.6. Data Analysis

Data were analyzed using a 2 × 4 factorial design to assess the effects of environmental conditions and Cr-yeast supplementation levels on physiological responses. Factor A (environmental conditions) included two levels: TN and HS. Factor B (Cr-yeast supplementation) included four dosage levels: 0, 0.2, 0.4, and 0.8 mg of Cr-yeast/kg of DMI, as previously described.

The experiment was analyzed using the model yijk = μ + τi + βj + γij + εijk, where yijk is the observed response variable, μ is the overall mean, τi is the fixed effect of the environmental condition (TN or HS), βj is the fixed effect of Cr-yeast dosage (0, 0.2, 0.4, or 0.8 mg/kg DMI), γij is the interaction effect between the two factors, and εijk is the residual error.

The eight treatment combinations were examined, and the data were analyzed using a two-way ANOVA to determine the main and interaction effects, followed by post hoc LSD Fisher test for significant effects in mean comparisons. The relative gene expression was calculated based on the 2^−ΔΔCt^ method [53] using *ywhaz* as a reference gene, and the results were expressed as fold change. Both gene expression and blood metabolite data were subjected to the same factorial analysis and post hoc testing. All statistical analyses were performed using GraphPad Prism v9.0 for Windows (La Jolla, CA, USA), with significance set at *p* < 0.05.

## 3. Results

### 3.1. Chromium Concentration in Blood

**Figure 1:**
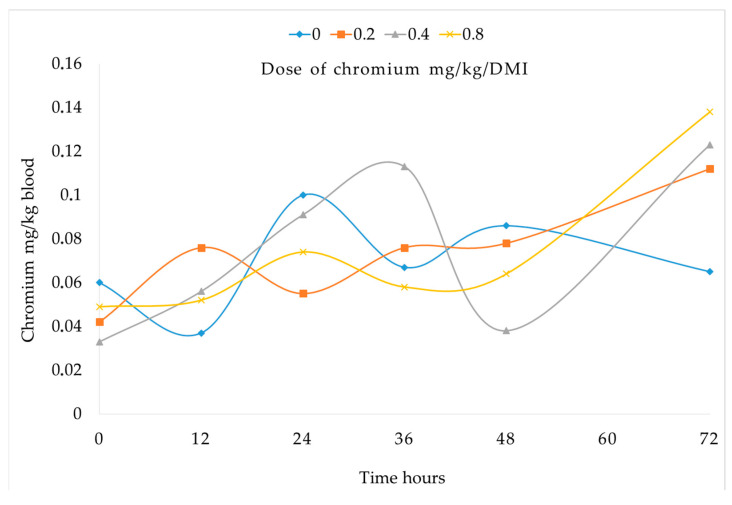
Chromium concentration in blood of sheep supplemented with different doses of organic chromium, samples were taken at 0, 12, 24, 36, 48, and 72 h after first dose.

The blood chromium concentration increased gradually with the ascending doses at the 12 h mark, with the 0.8 mg dose exhibiting a sustained elevation post-administration. This pattern was similar at 72 h post-treatment. In contrast, the control group (0.0 mg) maintained stable blood levels between 0.02 and 0.07 mg/L at 0 and 72 h, respectively (Figure 1).

### 3.2. Glucose Clearance in Blood

**Figure 2:**
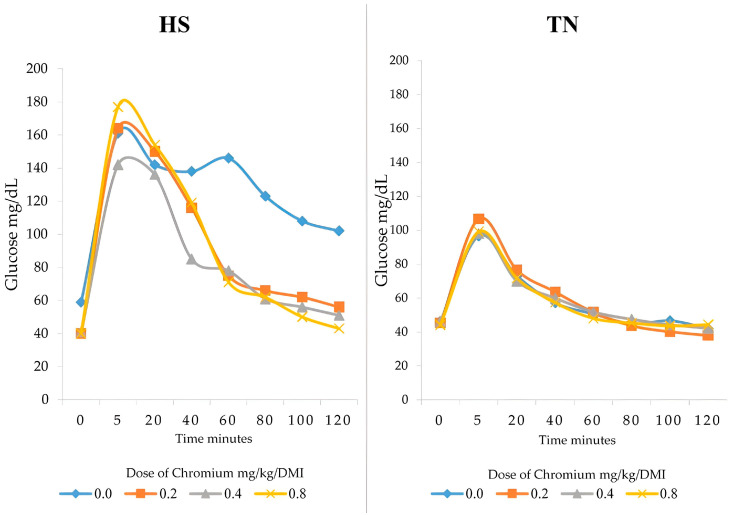
Blood glucose levels of lambs supplemented with different doses (0.0, 0.2, and 0.4, 0.8 mg/kg /DMI) of Cr-yeast under conditions of HS and TN.

The results of the glucose clearance test showed notable changes in the HS group. After 120 min, glucose levels in the control group remained around 102 mg/dL, while the other treatments exhibited a decrease in glucose levels as the Cr-yeast dosage increased. The lowest glucose level after 120 min was 43 mg/dL, observed with 0.8 mg of Cr-yeast (Figure 2 HS). In contrast, the TN group exhibited stable glucose levels across all treatments (Figure 2 TN).

### 3.3. Cortisol Levels

**Figure 3:**
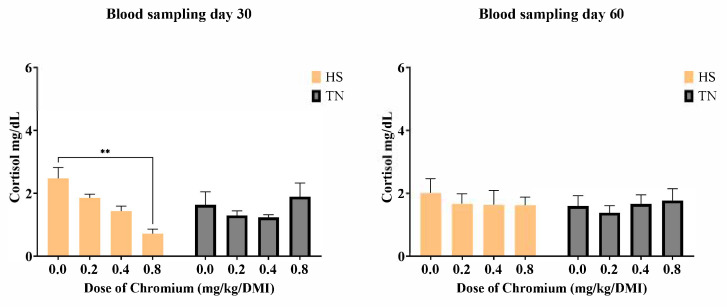
Blood cortisol levels of lambs supplemented with different doses (0.0, 0.2, and 0.4, 0.8 mg/kg/DMI) of Cr-yeast under conditions of HS and TN. Samples were taken on two different sampling days 30 and 60. ** indicates *p* < 0.01 between doses.

Figure 3 shows the effects of environmental conditions and Cr-yeast supplementation on plasma cortisol concentration. After 30 days, a significant interaction between environmental condition and Cr-yeast level was observed (*p* < 0.05). Under HS, cortisol levels were significantly lower in the group supplemented with 0.8 mg/kg compared to the non-supplemented group, with values returning to baseline by day 60. Under TN conditions, cortisol levels remained unchanged across all supplementation levels, indicating no significant effect of Cr-yeast in the absence of heat stress.

### 3.4. Gene Expression of hsp60 and hsp70

**Figure 4:**
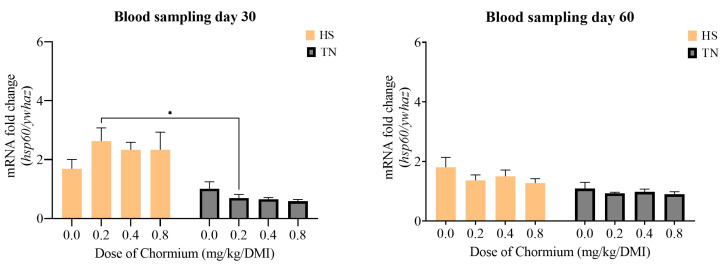
Relative expression of *hsp60* gene of serum from Katahdin sheep supplemented with Cr-yeast at HS and TN conditions, samples were taken on two different sampling days 30 and 60. * means *p* < 0.05 between doses.

**Figure 5:**
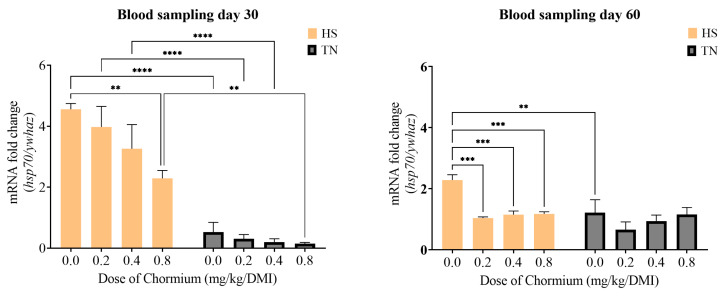
Relative expression of *hsp70* gene of serum from Katahdin sheep supplemented with Cr-yeast at HS and TN conditions, samples were taken on two different sampling days 30 and 60. ** indicates *p* < 0.01; *** indicates *p* < 0.001; **** indicates *p* < 0.0001 between doses.

A significant effect of temperature on *hsp60* transcript levels was observed on day 30, with the higher fold changes in HS at 0.2 mg Cr-yeast compared to TN conditions. By day 60, *hsp60* expression normalized across conditions (Figure 4). Conversely, on day 30, *hsp70* transcript levels were inversely correlated with Cr-yeast dosage, showing fold changes above 4 at 0.0 mg and close to 2 at 0.8 mg (Figure 5). Additionally, HS conditions increased *hsp70* transcripts compared to TN. By day 60, fold changes in HS conditions were stabilized from 0.2 mg onwards, indicating that transcript levels without supplementation remained higher than those at all Cr-yeast levels.

## 4. Discussion

Rising temperatures and prolonged heat waves increasingly compromise the welfare and productivity of small ruminants [6,7,8]. In this context, HS has become a critical concern due to its impact on metabolic, endocrine, and cellular functions. The present study aimed to evaluate whether Cr-yeast supplementation could mitigate some of these physiological and molecular disruptions in lambs subjected to chronic HS. Our findings demonstrate that supplementation with this organic form significantly improves chromium bioavailability, ameliorates impaired glucose clearance, and attenuates cellular stress markers, thereby contributing to enhanced thermotolerance in animals facing this challenge.

Under HS conditions, chromium excretion increases, making dietary supplementation necessary to mitigate stress-related effects [54,55,56]. Ngala et al. [57] emphasized its critical role in carbohydrate, lipid, and protein metabolism, and studies in rats, humans, and broilers have demonstrated its absorption in various tissues following supplementation [58,59,60]. However, a significant limitation in ruminants is the low bioavailability (<1%) of inorganic form [61,62]. In the present study, Cr-yeast supplementation increased blood chromium concentration within 72 h after a 0.8 mg dose (Figure 1), corresponding to an estimated bioavailability of 22.5% [63]. These results are consistent with previous findings; for instance, Hernandez-Garcia et al. [30] reported that the bioavailability of organic chromium is over ten times higher than that of inorganic forms, and Moreno-Camarena [64] reported a linear increase in levels of this trace mineral in the liver and bone of lambs supplemented with Cr-yeast, suggesting an improved bioavailability. These findings support the notion that the source of chromium greatly influences its uptake, with organic forms like Cr-yeast demonstrating superior bioavailability in ruminants compared to inorganic alternatives.

Among the effects caused by HS, impaired glucose clearance has been reported [35,65,66,67]. HS induces a response in the hypothalamic-pituitary axis, increasing cortisol levels, continuous glycogenolysis, and insulin resistance [68,69,70], which compromises glucose uptake by muscles and adipose tissue. Furthermore, elevated cortisol levels under HS are known to contribute to sustained hyperglycemia and impaired insulin signaling [55,56,71]. Chromium enhances insulin sensitivity through several mechanisms, including binding to chromodulin, activating insulin receptor tyrosine kinase, and promoting GLUT4 translocation [72,73,74]. As a component of the glucose tolerance factor (GTF), chromium also contributes to glucose regulation and cortisol reduction [56,75]. Our findings are consistent with these mechanisms: Cr-yeast supplementation under HS conditions reduced both serum glucose and cortisol levels, supporting the role of chromium in mitigating heat stress-associated metabolic dysfunction.

In addition, the behavior in the cortisol concentrations and the expression of *hsp60* and *hsp70* over the supplementation period suggests that Cr-yeast may influence the dynamics of cellular stress responses rather than achieving a full stabilization. This observation highlights the complexity of homeostatic regulation under chronic HS, where adaptative responses might be partial or influenced by the duration and intensity of exposure [76,77]. These findings are consistent with previous evidence indicating that oxidative or thermal challenges trigger a peak in *hsp* expression [78,79] followed by adaptation or resolution [69].

Notably, *hsp70* expression displayed a clear inverse relationship with Cr-yeast supplementation, which supports its role in modulating endocrine and molecular stress pathways. Since cortisol is a known inducer of hsp transcription [80], the pattern observed at day 30 may reflect the Cr-yeast capacity to alleviate cellular stress through improved insulin sensitivity and reduced oxidative damage [81,82], thereby indirectly reducing the intracellular burden that typically triggers *hsp* transcription [65,83]. The sustained elevation of *hsp70* at day 60 in the 0 mg group, despite some normalization in cortisol, suggests persistent subclinical stress or delayed cellular adaptation in the absence of chromium. This prolonged overexpression could lead to increased metabolic costs and negatively affect growth or immune responses [84].

Altogether, our results suggest that Cr-yeast supplementation not only mitigates systemic stress evidenced by lower cortisol and glucose but also attenuates molecular stress markers like *hsp70*, highlighting its potential role in enhancing thermotolerance and cellular recovery mechanisms under chronic heat stress conditions. While these findings are highly promising, future research could further explore the long-term adaptability of animals to chronic heat stress with supplementation, investigate the efficacy under more environmental conditions encountered in practical settings, and consider the potential influence of housing systems on animal welfare and stress responses.

## 5. Conclusions

An association was observed between thermal stress, cortisol synthesis, and the expression of serum mRNA for *hsp60* and *hsp70*. Supplementation with Cr-yeast modulated these biomarkers, indicating a reduction in stress levels.

Chromium also enhanced glucose clearance, lowering blood glucose concentrations in treated animals, suggesting improved insulin sensitivity under chronic heat stress.

Overall, Cr-yeast supplementation during HS shows strong potential to mitigate physiological stress responses. The 0.8 mg/kg DMI dose was the most effective, leading to the greatest reductions in cortisol and *hsp70* levels. Further research is needed to evaluate long-term effects and practical applications under different environmental and management conditions.

## Figures and Tables

**Table 1:** Ingredients composition on mixed diets (%).

Ingredients (as Fed Basis)	%
Soybean meal	9.20
Glycerol	4
Rice flour	21
Corn	15.20
Urea	0.25
Sulfur	0.05
Salt	0.30
Silage	50
Chemical composition of the basal diet used in the experiment DM (%)	
Nutrient	
Dry matter (DM)	49.61
Organic matter (OM)	89.17
Ash	10.82
Crude protein (CP)	12.12
Ethereal extract (EE)	7.65
Neutral detergent fiber (NDF)	40.90
Acid detergent fiber (ADF)	11.03
Non-structural carbohydrates (NSC)	28.50

Cr-yeast levels were administered independently of diet.

**Table 2:** Primer sequences for amplification of *hsp60* and *hsp70* by qPCR.

Gene	Gene Name	Accession Number	Sequence	Size of the Amplicon (bp)
*ywhaz*	3-monooxygenase/tryptophan 5-monooxygenase activation protein, zeta polypeptide	NM_001267887.1	F-AGCAGGCTGAGCGATATGAT	180
			R-TCTCAGCACCTTCCGTCTTT	
*hsp60*	Heat shock protein 60	XM_027965061.1	F-ACTGGCTCCTCATCTCACTC	147
			R-TGTTCAATAATCACTGTCCTTCC	
*hsp70*	Heat shock protein 70	NM_001267874.1	F-CGGAGAAGGACGAGTTTGAG	165
			R-AATCCACCTCCTCAATGGTG	

## Data Availability

The data presented in this study are available on request from the corresponding author.
